# Supplementary material for: Edge states and skyrmion dynamics in nanostripes of frustrated magnets
Source: Nat Commun. 2017 Feb 27;8:14394. doi: 10.1038/ncomms14394 (PMC5333371; doi:10.1038/ncomms14394)
Supplement: Supplementary Information — Supplementary Note 1 and Supplementary References. [file ncomms14394-s1.pdf]

Supplementary Note 1: Skyrmion center-of-mass and helicity dynamics induced by an electric current

We start by rewriting the Landau-Lifshitz-Gilbert equation in the form,

$$\dot{\mathbf{m}} = \mathbf{m} \times \frac{\partial E}{\partial \mathbf{m}} + \alpha\, \mathbf{m} \times \dot{\mathbf{m}} + (\mathbf{j} \cdot \nabla)\mathbf{m} - \beta\, \mathbf{m} \times (\mathbf{j} \cdot \nabla)\mathbf{m}. \tag{1}$$

Equations for the set of skyrmion collective coordinates $\{\xi_i(t)\}$, which includes the center-of-mass coordinates, x and y, are obtained by multiplying both parts of Supplementary Eq. (1) with $\mathbf{m} \times \frac{\partial \mathbf{m}}{\partial \xi_i}$ and integrating over the spatial coordinates [1]:

$$\alpha \Gamma_{ij} \dot{\xi}_j - G_{ij} \dot{\xi}_j = -\frac{\partial E}{\partial \xi_i} + j_x \Gamma_{ix} - \beta G_{ix} j_x, \tag{2}$$

where $\Gamma_{ij} = \Gamma_{ji} = \int d^2 r\, \frac{\partial \mathbf{m}}{\partial \xi_i} \cdot \frac{\partial \mathbf{m}}{\partial \xi_j}$ and $G_{ij} = -G_{ji} = \int d^2 r\, \mathbf{m} \cdot \frac{\partial \mathbf{m}}{\partial \xi_i} \times \frac{\partial \mathbf{m}}{\partial \xi_j}$.

Here we only consider two pairs of collective coordinates: (x, y) and (χ, M_z), where χ is the skyrmion chirality and $M_z = \int d^2 r\, (m_z + 1)$ is the z-component of the skyrmion magnetic moment counted from the magnetic moment of the uniform ferromagnetic state with $m_z = -1$. The variables forming pairs are mutually conjugated, i.e. only $G_{xy} = -G_{yx}$ and $G_{\chi M_z} = -G_{M_z \chi}$ are nonzero:

$$G = G_{xy} = 4\pi Q, \tag{3}$$

where Q is the skyrmion topological charge and

$$G_{\chi M_z} = 1. \tag{4}$$

The last equation follows from the form of the Berry phase action [2], $-\int dt d^2 r\, m_z \dot{\varphi}$, which gives $-\int dt M_z \dot{\chi}$, if the time derivative of the azimuthal angle $\varphi(\mathbf{r}, t)$ describing the direction of $\mathbf{m}$ equals $\dot{\chi}$ for all spins. The M_z-variable is essentially a breathing mode, *i.e.* the variation of the skyrmion radius. This can be understood by considering a cylindrical domain (magnetic bubble) with skyrmion topology, in which case the helicity angle, χ, describing the rotation from the Néel to Bloch domain wall, is conjugated to the radius of the cylindircal domain [3].

By symmetry, the only nonzero Γ_{ij} coefficients are: $\Gamma = \Gamma_{xx} = \Gamma_{yy}$, $\Gamma_{\chi\chi} = \int d^2 r\, (1 - (m_z)^2)$

and $\Gamma_{M_z M_z}$, so that the system of Supplementary Eqs. (2) reduces to

$$\begin{cases} \alpha\Gamma\dot{X} - G\dot{Y} &= j_x\Gamma, \\ G\dot{X} + \alpha\Gamma\dot{Y} &= -\frac{\partial U}{\partial y} + \beta j_x G, \\ \alpha\Gamma_{\chi\chi}\dot{\chi} - \delta\dot{M}_z &= -\frac{\partial U}{\partial \chi}, \\ \dot{\chi} + \alpha\Gamma_{M_z M_z}\delta\dot{M}_z &= -K\delta M_z, \end{cases} \tag{5}$$

where $\delta M_z = M_z - M_z^{(0)}$ is the deviation of the skyrmion magnetic moment from its value for a free skyrmion. This deviation is relatively small because of the large skyrmion rigidity, which justifies the use of harmonic approximation for M_z-dependence of the skyrmion energy, $\frac{1}{2}K(\delta M_z)^2$, in Supplementary Eq. (5). Furthermore, the dissipation term, $\alpha\Gamma_{M_z M_z}\delta\dot{M}_z$, in the last equation of Supplementary Eq. (5) can be neglected, which gives a second-order equation of motion for χ,

$$\mathcal{M}\ddot{\chi} + \alpha\Gamma_{\chi\chi}\dot{\chi} = -\frac{\partial U}{\partial \chi}, \tag{6}$$

$\mathcal{M} = K^{-1}$ being the effective mass for the chirality dynamics.

Supplementary References

---

[1] Tretiakov, O., A., Clarke, D., Chern, G.-W., Bazaliy, Ya. B. and Tchernyshyov, O., *Dynamics of Domain Walls in Magnetic Nanostrips*, Phys. Rev. Lett. **100**, 127204 (2008).

[2] Zang, J., Mostovoy, M., Han, J. H. and Nagaosa, N. Dynamics of skyrmion crystals in metallic thin films. *Phys. Rev. Lett.* **107**, 136804 (2011).

[3] Malozemoff, A. P. and Slonczewski, J. C. *Magnetic Domain Walls in Bubble Materials* (New York: Academic, 1979).
